# Optimizing source-sink relationships and photoprotective mechanisms in wheat to enhance photosynthetic efficiency and nitrogen management

**DOI:** 10.3389/fpls.2025.1660834

**Published:** 2025-09-18

**Authors:** Siqi Zhang, Xiaohui Li, Xinyu Wang, Jie Zhang, Guoqiang Li, Zhongwei Tian

**Affiliations:** ^1^ Institute of Agricultural Information Technology, Henan Academy of Agricultural Sciences, Zhengzhou, China; ^2^ College of Plant Protection, Henan Agricultural University, Zhengzhou, China; ^3^ Key Laboratory of Crop Physiology Ecology and Production Management, Ministry of Agriculture/College of Agriculture, Nanjing Agricultural University, Nanjing, China; ^4^ Huanghuaihai Key Laboratory of Intelligent Agricultural Technology, Ministry of Agriculture and Rural Areas, Zhengzhou, China

**Keywords:** wheat, source sink relationship, photosynthetic capacity, photoprotection, nitrogen management

## Abstract

Although the source-sink relationship is an intrinsic factor affecting crop photosynthesis, the response of different source-sink types to nitrogen (N) availability in photoprotection and photosynthetic efficiency is still unclear. This study investigates the physiological mechanisms underlying differences in photosynthetic capacity and photoprotection in wheat with different source-sink relationships under varying N levels. Field experiments revealed that compared to sink-limited wheat (YM1), source-limited wheat (YM25 and ZM27) maintained higher chlorophyll content, maximum net photosynthetic rate (*Pn_max_*), and light energy utilization efficiency, and alleviated photoinhibition by reducing non-photochemical quenching (NPQ) and increasing the proportion of cyclic electron flow (CEF). Additionally, source-limited wheat flag leaves had higher carotenoid content and soluble protein content and stronger antioxidant capacity, which enabled them to scavenge reactive oxygen species, reduce membrane lipid peroxidation, and delay leaf senescence. N fertilization significantly improved wheat’s photosynthetic capacity and light energy utilization efficiency, alleviating photoinhibition. Source-limited wheat can still maintain the integrity of the photosynthetic apparatus under low N conditions by enhancing photoprotective mechanisms, showing stronger environmental adaptability. Therefore, proper N fertilization and optimization of source-sink relationships help improve wheat’s photosynthetic capacity and yield potential.

## 1 Introduction

Photosynthesis is a complex physiological and biochemical process that includes CO_2_ transport, photosynthetic pigment synthesis, photosynthetic electron transfer, light energy conversion, and the Calvin cycle, among others (Liu et al., 2023). Photosynthesis in C3 crops includes the light reaction and dark reaction. The light reaction occurs in a series of pigment-protein complexes and electron carriers on the thylakoid membrane, which have specific spatial arrangements and structural changes. Chlorophyll absorbs light energy and converts it into electrical energy, transferring electrons through the electron transport chain to NADPH+, and coupling with photophosphorylation to generate ATP for the dark reaction. The dark reaction, also known as the Calvin cycle, occurs in the stroma of the chloroplast, with the key enzyme being Rubisco, whose carboxylation ability is a limiting factor in photosynthetic capacity (Ślesak and Ślesak, 2021). Only when the processes of photosystem structure, light energy capture, electron transfer, Rubisco carboxylation, and photosynthetic product output are coordinated and interact can photosynthetic efficiency be maximized (Miri, 2009).

The source-sink structure is an intrinsic factor that affects crop photosynthesis and is also one of the main quality characteristics of high photosynthetic efficiency crop populations (Fernie et al., 2020). There are significant differences in net photosynthetic rate (*Pn*) among wheat cultivars with source-sink relationships. Studies have shown that large-spike cultivars with high sink strength have higher photosynthetic activity in non-leaf organs than small-spike cultivars (Wang et al., 2001). Zhang et al. (2024a) proposed that source-limited wheat is more rational than sink-limited wheat, as they have larger leaf area, longer leaf area duration, and stronger photosynthetic performance, while sink-limited wheat exhibits lower photosynthetic capacity. Studies generally suggest that when source activity is greater or sink demand decreases (usually due to insufficient sink activity), source-sink imbalance occurs, leading to the accumulation of soluble carbohydrates in source leaves, which downregulates photosynthesis-related genes and accelerates leaf senescence (Burnett et al., 2016). On the other hand, insufficient source activity can lead to early leaf senescence, hindering the accumulation of photosynthetic products in the leaves (Krahmer et al., 2018). It’s widely recognized that modern wheat cultivars’ sinks can still boost yields. However, past breeding has shifted from sink limitation to more source limitation, and the source will limit future yield growth (Zhang et al., 2016). Thus, optimizing the balance between source and sink organs is crucial for ensuring higher yield. Understanding the regulatory mechanisms of the source-sink relationship in photosynthesis provides a theoretical basis for improving the source performance of crop yield.

During photosynthesis, the photosynthetic apparatus in leaves can be damaged when the absorbed light energy exceeds its maximum light energy utilization capacity, leading to a decrease in photosynthetic efficiency and causing photoinhibition (Djanaguiraman et al., 2020). Non-photochemical quenching (NPQ) is a key photoprotective mechanism by which excess light energy is dissipated as heat, protecting the photosynthetic machinery from damage (Lu et al., 2022). However, excessive NPQ can reduce quantum efficiency, which in turn may negatively affect crop yields. Imbalances in the source-sink relationship have been shown to lead to an accumulation of sucrose in source leaves, suppressing the expression of photosynthesis-related proteins, reducing electron transfer, and increasing heat dissipation, thereby exacerbating photoinhibition (Muller et al., 2014). When excess light energy is available, the enhanced electron transport pathway using oxygen as an electron acceptor induces the production of reactive oxygen species (Zhang et al., 2024b). Reactive oxygen species can cause lipid peroxidation, damage the membrane system, and affect normal leaf metabolism (Shimakawa et al., 2022). Superoxide dismutase (SOD), catalase (CAT), and peroxidase (POD) in plants are important components of the reactive oxygen species scavenging system. They effectively prevent the accumulation of high concentrations of oxygen and protect against lipid peroxidation (Ewald, 2018; Liu et al., 2025). Some studies suggest that a higher sink-source ratio can improve leaf *Pn*, significantly increase SOD activity, and slow the increase in malondialdehyde content (Wada and Wada, 1991). Other studies show that changing the source-sink ratio, with a decrease in sink volume, increases leaf chlorophyll content, *Pn*, and leaf SOD, CAT, and POD activity (Jiang et al., 2004). These seemingly contradictory findings may arise from differences in cultivars, growth conditions, manipulation methods, or measurement techniques. Whether changing the source-sink relationship can alleviate photoinhibition and improve wheat’s antioxidant capacity still requires further investigation. Therefore, a systematic comparison of well-defined source- and sink-limited cultivars under controlled nitrogen (N) levels is necessary to clarify these physiological mechanisms.

While considerable research has been conducted to explore the physiological mechanisms of the source-sink relationship, a comprehensive understanding of its regulatory impact on photosynthesis remains incomplete. To determine the variation patterns of photosynthetic traits in wheat with different source-sink relationships, this study selected three wheat cultivars with source-sink relationships and set two N levels to explore the differences in photosynthetic capacity, light energy distribution between photosystem I (PSI) and photosystem II (PSII), and photoprotection abilities. The aim is to clarify the physiological mechanisms underlying differences in photosynthetic capacity and photoprotection in wheat cultivars with different source-sink relationships under varying N levels. Ultimately, this research seeks to contribute to the development of strategies to optimize source-sink relationships for improving wheat yield potential.

## 2 Material and methods

### 2.1 Experiment design

**Figure 1:**
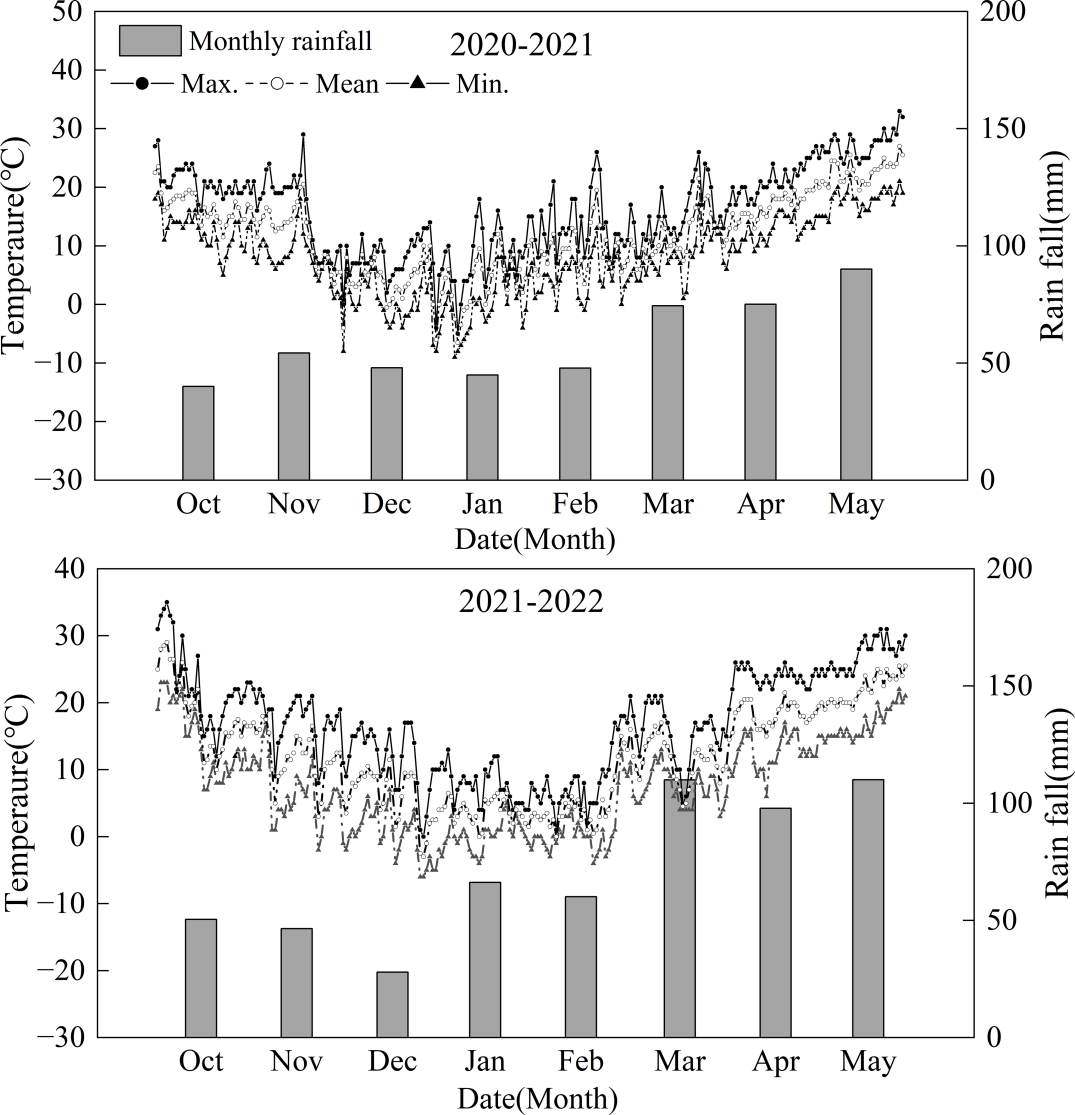
Daily maximum (max.) and minimum (min.) and mean temperatures and monthly rainfall for the two wheat growing seasons (2020–2021 and 2021-2022).

The experiment was conducted at the experimental base of Nanjing Agricultural University (32°24’N, 118°9’E) in Nanjing, China, during the 2020/2021 and 2021/2022 growing seasons. A field experiment was conducted, selecting three different wheat cultivars: Yangmai 1 (YM1, southern cultivar, sink-limited wheat), Yangmai 25 (YM25, southern cultivar, source-limited wheat), and Zhoumai 27 (ZM27, northern cultivar, source-limited wheat), which was studied in our previous work (Zhang et al., 2024a). According to the response of different cultivars to source and sink manipulations, YM25 and ZM27 had a higher sink–source ratio, manifesting as a source-limited cultivar. YM1 has a lower sink–source ratio, manifesting as a sink-limited cultivar. Two N fertilizer treatments were set, with N application rates of 240 kg·hm^-2^ and 120 kg·hm^-2^, referred to as N240 and N120, respectively. A completely randomized block design was used, with N fertilizer as the main factor and variety as the secondary factor. The basic seeding was 2.25×10^6^ seeds ha^−1^. The plot size was 3.0 m×3.0 m (9m^2^) with 12 rows and a spacing of 0.25 m between rows. 50% of the N fertilizer and all phosphorus (P_2_O_5_, 150 kg·hm^-2^) and potassium fertilizer (K_2_O, 150 kg·hm^-2^) were used as base fertilizer, with the remaining 50% of N applied at the jointing stage. Soil samples at a depth of 0 to 25 cm were found to have 20.44 g·kg^–1^ organic matter, 1.35 g·kg^–1^ total N content, 65.22 mg·kg^–1^ available N content, 18.49 mg·kg^–1^ available P_2_O_5_ content, and 109.44 mg·kg^–1^ rapidly available K in 2020–2021 and 26.98 g·kg^–1^ organic matter, 1.40 g·kg^–1^ total N content, 68.22 mg·kg^–1^ available N content, 12.37 mg·kg^–1^ available P_2_O_5_ content, and 93.76 mg·kg^–1^ rapidly available K in 2021–2022 The sowing dates were November 2 in 2020, and November 13 in 2021, and the harvesting dates were May 24, 2021, and May 26, 2022. Seedlings were thinned at the three-leaf stage, and stakes were provided to prevent lodging for lodging-prone cultivars, ensuring stable yield performance for all cultivars. Management followed local cultivation practices, with timely pest and disease control. The average temperature and cumulative precipitation meteorological conditions during wheat growth were calculated according to the meteorological data provided by the local weather station and are shown in **Figure 1**. The climate is humid and warm, with an annual rainfall of 476.60 mm and 568.76 mm during 2020/2021 and 2021/2022 growing seasons, respectively. The range of monthly average temperatures in the region was 4-22°C and 4-22°C during 2020/2021 and 2021/2022 growing seasons, respectively. Meteorological conditions during the wheat growing seasons were typical for the region, with no extreme events noted that would significantly impact the treatments differently.

### 2.2 Determination of photosynthetic pigment content

Samples of the marked flag leaves were taken at 0, 10, 20, and 30 days after anthesis (DAA). The determination of photosynthetic pigment content was carried out using the acetone extraction method (Lichtenthaler and Buschmann, 2001). A 0.05 g fresh wheat leaf sample was taken, chopped, and mixed. Acetone and anhydrous ethanol were mixed in equal proportions, and 20 mL of the mixed extraction solution was added. The mixture was placed in a 40 °C constant temperature incubator and extracted in a glass test tube for about 24 hours in the dark. The absorbance values at 663 nm, 645 nm, and 470 nm were measured using a UV spectrophotometer. Chlorophyll a, chlorophyll b, total chlorophyll, and carotenoids in the leaves were calculated using the following Equations 1–4:


(1) $$\text{Chl a}(\text{mg}\cdot {\text{g}}^{-1})=(12.25\times {\text{A}}_{663}-2.79\times {\text{A}}_{645})\times \text{V}\;/\;(1000\times \text{W})$$

(2) $$\text{Chl b}\;(\text{mg}\cdot {\text{g}}^{-1})=(21.50\times {\text{A}}_{645}-5.10\times {\text{A}}_{663})\times \text{V}/(1000\times \text{W})$$

(3) $$\text{Total chlorophyll}(\text{mg}\cdot {\text{g}}^{-1})=\text{Chl a}+\text{Chl b}$$

(4) $$\begin{array}{c} \text{Carotenoids}(\text{mg}\cdot {\text{g}}^{-1})=[(1000\times {\text{A}}_{470}-1.82\times \text{Chl a}-85.02\times \text{Chl b})/198]\times \\ \text{V}/(1000\times \text{W}) \end{array}$$

where A is the absorbance at the specified wavelength, V is the volume of extract (mL), and W is the fresh weight of the sample (g).

### 2.3 Measurement of light response curve

The light response curve was measured using the LI-6400 portable photosynthesis system (Li-Cor Inc., USA) at 0, 10, and 20 DAA. The photosynthetically active radiation (PAR) was set to 1200 μmol·m^-2^·s^-1^, with the CO_2_ concentration in the leaf chamber set to about 400 μmol·CO_2_·L^-1^, the leaf temperature set to 25.0 ± 0.5 °C, and the relative humidity in the leaf chamber set to about 65 ± 5%. Three uniform and healthy wheat flag leaves were selected from each plot, and measurements were taken simultaneously using three photosynthesis systems during 09:00-11:00 a.m. on clear days without clouds. Before measurements, the leaves were placed in the leaf chamber with PAR set to 1200 μmol·m^-2^·s^-1^ for 10 min. The PAR gradient was set to 0, 50, 100, 150, 200, 400, 600, 800, 1000, and 1200 μmol·m^-2^·s^-1^. After the measurements, a curve was plotted with *Pn* as the vertical axis and light intensity as the horizontal axis. The initial slope of the curve (when PPDF < 200 μmol·m^-2^·s^-1^) represented the apparent quantum yield (*Φ_CO2_*). The maximum net photosynthetic rate (*Pn_max_*) was represented by the peak of the light response curve (Li et al., 2009), with data recorded after the system reached equilibrium (2–3 min).

### 2.4 Determination of photoprotective mechanism-related parameters

The measurements were conducted at 0, 10, 20, and 30 DAA using a Dual-PAM-100 dual-channel chlorophyll fluorometer (Heinz Walz, Effeltrich, Germany), controlled by the standard protocol of Dual-PAM software v1.19. Before measurements, plants were dark-adapted for 30 min to determine the minimum fluorescence (F_o_) and maximum fluorescence (F_m_). Actinic light at 1200 μmol·m^-2^·s^-1^ was applied for 10 min until steady-state fluorescence was reached; subsequently, a saturating pulse light intensity of 10000 μmol·m^-2^·s^-1^ was applied to measure, including steady-state fluorescence (F_s_), light-adapted maximum fluorescence (F_m′_), maximum PSI oxidation (P_m′_), and quantum yields of energy conversion: PSI effective quantum yield (Y(I)), PSII effective quantum yield (Y(II)), quantum yield of non-photochemical quenching due to acceptor-side limitation in PSI (Y(NA)), quantum yield of non-photochemical quenching due to donor-side limitation in PSI (Y(ND)). Electron transport rates (ETR(I) and ETR(II)) and cyclic electron flow (CEF) were subsequently calculated. Additionally, thermal dissipation parameters were quantified, including NPQ, photoprotective mechanism-associated non-photochemical quenching quantum yield (*Φ_NPQ_*), and non-photochemical quenching quantum yield unrelated to photoprotection (*Φ_NO_*). The calculations followed established formulas Equations 5–10 (Trebst, 1980; Öquist and Chow, 1992).


(5) ETR(I)=Y(I)×PAR×0.85×0.5

(6) ETR(II)=Y(II)×PAR×0.85×0.5

(7) CEF=ETR(I)−ETR(II)

(8) NPQ=(Fm−Fm')/Fm

(9) ΦNO=1/NPQ+1+QL×(Fm/Fo−1)

(10) ΦNPQ=1−ФPSII−ΦNO

### 2.5 Determination of soluble protein content

Marked flag leaves were sampled at 0, 10, 20, and 30 DAA. Soluble protein content was determined using the Coomassie Brilliant Blue G-250 method (Snyder and Desborough, 1978). A 0.1 g fresh sample was homogenized with 1 mL phosphate-buffered saline (PBS, pH 7.2). The homogenate was centrifuged at 4 °C and 4000 × g for 10 min. Then, 0.1 mL of the supernatant was mixed with 5 mL of Coomassie Brilliant Blue reagent. After thorough mixing, the absorbance was measured at 595 nm using a UV spectrophotometer. Soluble protein concentration was then calculated accordingly.

### 2.6 Determination of superoxide anion and malondialdehyde contents

Flag leaves were sampled at 0, 10, 20, and 30 DAA. Fresh wheat leaf samples (0.5 g) were homogenized in 3 mL of enzyme extraction buffer containing HEPES buffer (pH 7.8), 20% (v/v) glycerol, 1 mM ascorbic acid (AsA), 1 mM EDTA, 5 mM MgCl_2_, 1 mM reduced glutathione (GSH), and 1 mM dithiothreitol (DTT). The homogenate was centrifuged at 10,000 × g for 20 min at 4 °C, and the supernatant was collected as the crude enzyme extract.

The production rate of superoxide anion (O_2_^-^) in leaves was determined using the hydroxylamine method (Pageau et al., 2006). A 0.5 mL aliquot of crude enzyme extract was mixed with 0.5 mL HEPES buffer (pH 7.8) and 1 mL of 1 mM hydroxylamine hydrochloride, and incubated at 25 °C for 1 h. Subsequently, 1 mL of α-naphthylamine and 1 mL of p-aminobenzenesulfonic acid were added, and the mixture was left to stand for 20 min at 25 °C. Absorbance was measured at 530 nm using a UV spectrophotometer.

Malondialdehyde (MDA) content in the leaves was determined using the thiobarbituric acid (TBA) colorimetric method (Kramer et al., 2004). A 2 mL sample of crude enzyme extract was mixed with 4 mL of trichloroacetic acid-thiobarbituric acid (TCA-TBA) reagent, and then heated in a boiling water bath for 20 min. After centrifugation at 4000 × g for 10 min, the supernatant was collected and its absorbance was measured at 450 nm, 532 nm, and 600 nm using a UV spectrophotometer.

### 2.7 Determination of antioxidant enzyme activity

Flag leaves were sampled at 0, 10, 20, and 30 DAA. Fresh wheat leaf samples (0.5 g) were homogenized with 5 mL of enzyme extraction buffer containing 5 mM phosphate-buffered saline (PBS, pH 7.5), 1 mL of 5.0% (w/v) polyvinylpolypyrrolidone (PVPP), and 1 mL of 0.1 mM ethylenediaminetetraacetic acid (EDTA). The homogenate was centrifuged at 12,000 × g for 2 min at 4 °C, and the supernatant was collected as the crude enzyme extract.

SOD activity was determined using the nitroblue tetrazolium (NBT) method as described by Tan et al. (2008). A 0.05 mL aliquot of crude enzyme extract was added to a reaction mixture containing 0.3 mL of 100 μM EDTA-Na_2_, 0.3 mL of 130 mM methionine, 0.3 mL of 20 μM riboflavin, and 0.3 mL of 50 μM NBT. The mixture was exposed to 4000 lx light for 15 min. A non-illuminated sample served as the blank control. Absorbance at 560 nm was measured using a UV spectrophotometer, and SOD activity was calculated accordingly.

POD activity was determined using the guaiacol method as described by Zeng et al. (2017). A 10 μL aliquot of crude enzyme extract was added to a reaction mixture containing 0.3 mL of 0.2 M phosphate buffer (PBS, pH 6.0), 0.02 mL of 0.05 M guaiacol, and 0.03 mL of 30% hydrogen peroxide. After thorough mixing, absorbance at 470 nm was measured using a UV spectrophotometer to calculate POD activity.

CAT activity was determined using the ultraviolet absorption method, as described by Hadwan (2018). A 0.1 mL aliquot of crude enzyme extract was mixed with 1.9 mL of phosphate buffer (PBS, pH 7.0) and 1 mL of 0.075% hydrogen peroxide. After thorough mixing, the absorbance at 240 nm was measured using a UV spectrophotometer to calculate CAT activity.

The activity of ascorbate peroxidase (APX) was determined using the ascorbate method (Wu et al., 1998). A 0.1 mL aliquot of crude enzyme extract was mixed with 1 mL of 50 mM phosphate buffer (PBS, pH 7.0), 1 mL of 0.3 mM ascorbic acid, 2 mM EDTA, and 20 μL of 30% H_2_O_2_. Absorbance at 290 nm was measured using a UV spectrophotometer to calculate APX activity.

### 2.8 Statistical analysis

Statistical analysis was calculated using SPSS 19.0 (SPSS Inc., Chicago, USA) and included a one-way analysis of variance (ANOVA). For data presented in charts, Duncan’s test was conducted, with statistical significance accepted at *P < 0.05*. All graphs presented were produced using Origin 2025 (OriginLab, Northampton, MA, USA).

## 3 Results

### 3.1 Photosynthetic pigment content

**Figure 2:**
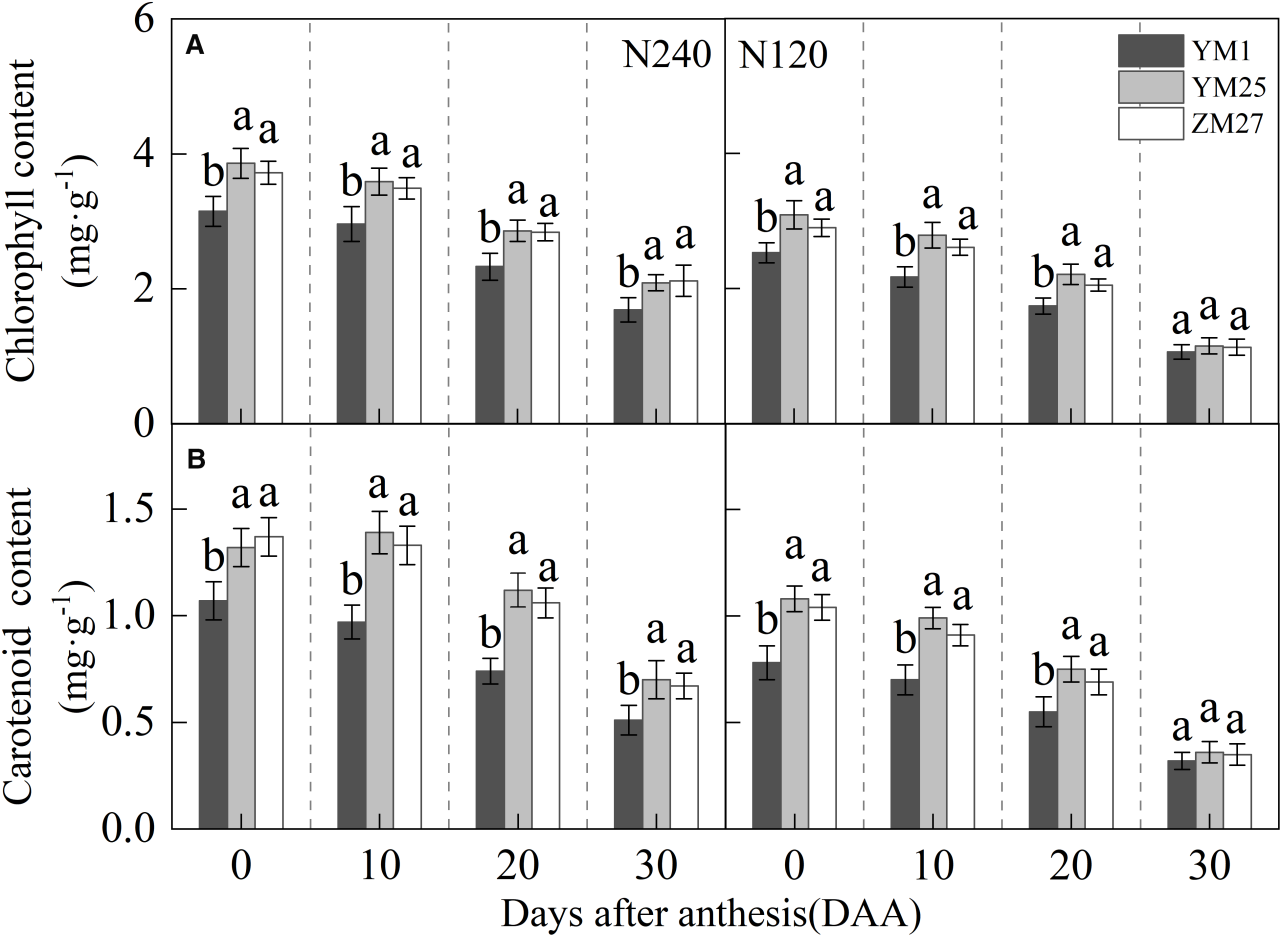
Differences of chlorophyll content **(A)** and carotenoid content **(B)** in flag leaves of wheat with different source-sink relationships. Notes: N240 and N120 respectively represent nitrogen application rates of 240 and 120 kg·hm-2. Each value represents the mean ± SD of three biological replicates. Different lowercase letters indicate statistically significant differences between treatments (*P < 0.05*) according to LSD test. Error bars indicate SD.

The chlorophyll and carotenoid contents in flag leaves gradually declined with days after anthesis, and decreased under lower N levels (**Figures 2A, B**). Both YM25 and ZM27 exhibited significantly higher chlorophyll and carotenoid contents than YM1 during 0–20 DAA, regardless of N treatment. No significant differences in pigment content were observed between YM25 and ZM27. The low N treatment (N120) significantly reduced chlorophyll and carotenoid levels in the flag leaves of all cultivars, highlighting the importance of N for maintaining photosynthetic pigment stability. These findings suggest that YM25 and ZM27, which are source-limited wheat, maintain better light energy absorption and utilization efficiency, even under reduced N conditions.

### 3.2 Maximum net photosynthetic rate and apparent quantum efficiency

**Figure 3:**
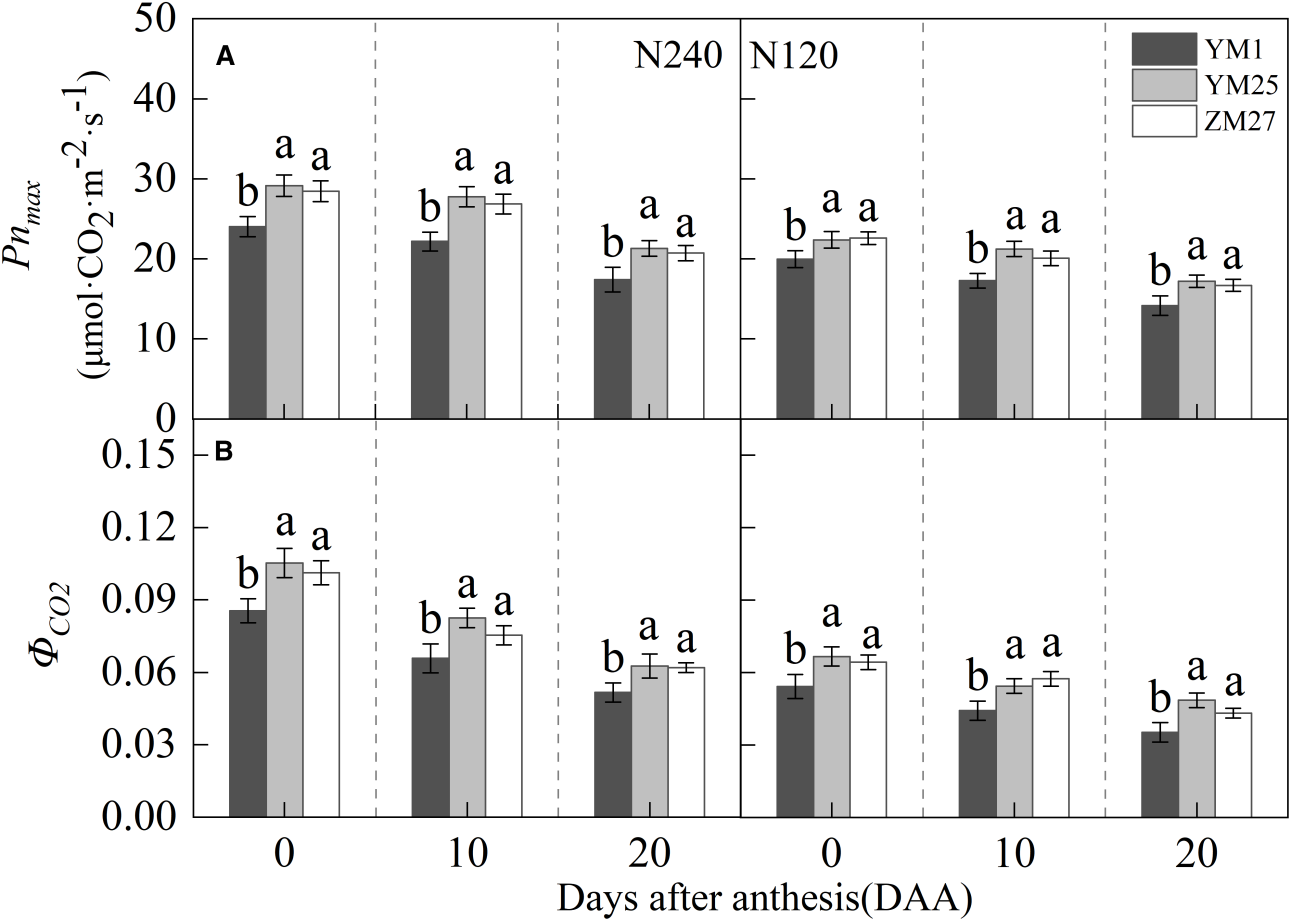
Differences of max net photosynthetic rate (*Pn_max_*) **(A)** and apparent quantum efficiency (*Φ_CO2_*) **(B)** in flag leaves of wheat with different source-sink relationships. Notes: Each value represents the mean ± SD of three biological replicates. Different lowercase letters indicate statistically significant differences between treatments (*P < 0.05*) according to LSD test. Error bars indicate SD.

The flag leaf *Pn_max_* and *Φ_CO2_* gradually decreased with days after anthesis and decreased under lower N levels (**Figures 3A, B**). Under both N240 and N120 conditions, YM25 and ZM27 flag leaf *Pn_max_* and *Φ_CO2_* were significantly higher than YM1 during 0–20 DAA. No significant differences were observed between YM25 and ZM27. Low N conditions significantly reduced the flag leaf *Pn_max_* and *Φ_CO2_* in all cultivars. These results indicate that the source-limited wheat has superior photosynthetic capacity, demonstrating enhanced ability to capture and utilize solar radiation effectively. In contrast, YM1, a sink-limited wheat, exhibited lower photosynthetic efficiency, particularly under low N conditions.

### 3.3 PSII heat dissipation and light protection-related/unrelated heat dissipation

**Figure 4:**
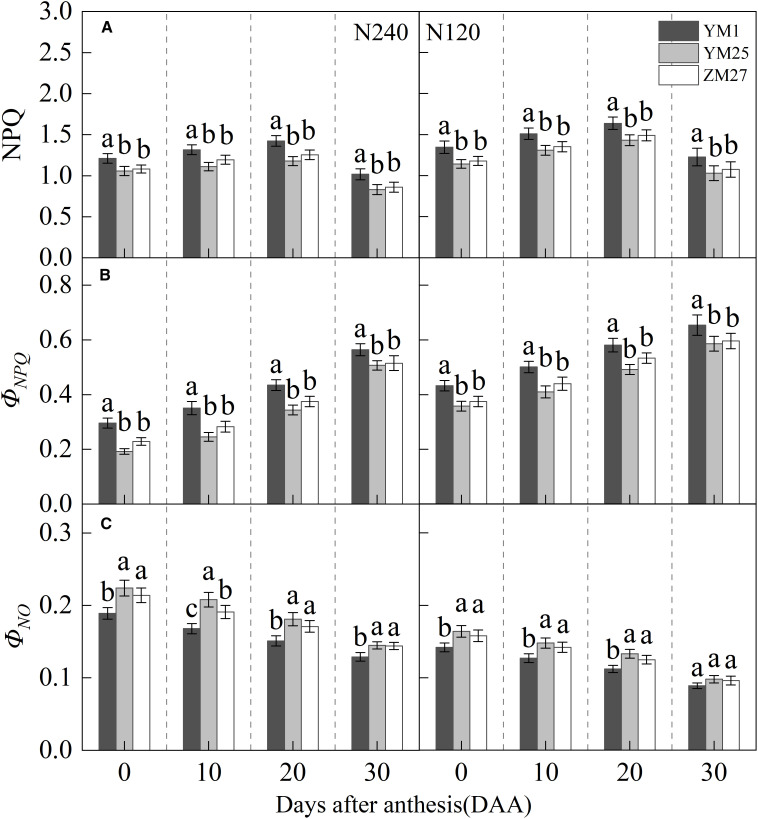
Differences of non-photochemical quenching (NPQ) **(A)**, photoprotective mechanism-associated non-photochemical quenching quantum yield (*Φ_NPQ_*) **(B)**, and non-photochemical quenching quantum yield unrelated to photoprotection (*Φ_NO_*) **(C)** in flag leaves of wheat with different source-sink relationships. Notes: Each value represents the mean ± SD of three biological replicates. Different lowercase letters indicate statistically significant differences between treatments (*P < 0.05*) according to LSD test. Error bars indicate SD.

NPQ, a key indicator of heat dissipation, initially increased and then decreased with days after anthesis, reaching a maximum value at 20 DAA, and increased under lower N levels (**Figure 4A**). *Φ_NPQ_* gradually increased with days after anthesis and decreased with reduced N application (**Figure 4B**). Additionally, *Φ_NO_* gradually decreased with days after anthesis and increased with decreasing N application (**Figure 4C**). Under both N treatments, YM25 and ZM27 NPQ and *Φ_NPQ_* were significantly lower than YM1 during 0–20 DAA, while *Φ_NO_* was significantly higher than YM1 during the same period. No significant differences were observed between YM25 and ZM27. Under low N conditions, flag leaf NPQ and *Φ_NPQ_* significantly increased, while *Φ_NO_* significantly decreased in all cultivars. These results suggest that the source-limited wheat (YM25 and ZM27) can dissipate excess light energy more efficiently, thereby protecting the photosynthetic apparatus from photoinhibition. However, under low N conditions, the degree of photoinhibition increased, resulting in enhanced heat dissipation and greater light-induced damage.

### 3.4 PSI non-photochemical energy dissipation

**Figure 5:**
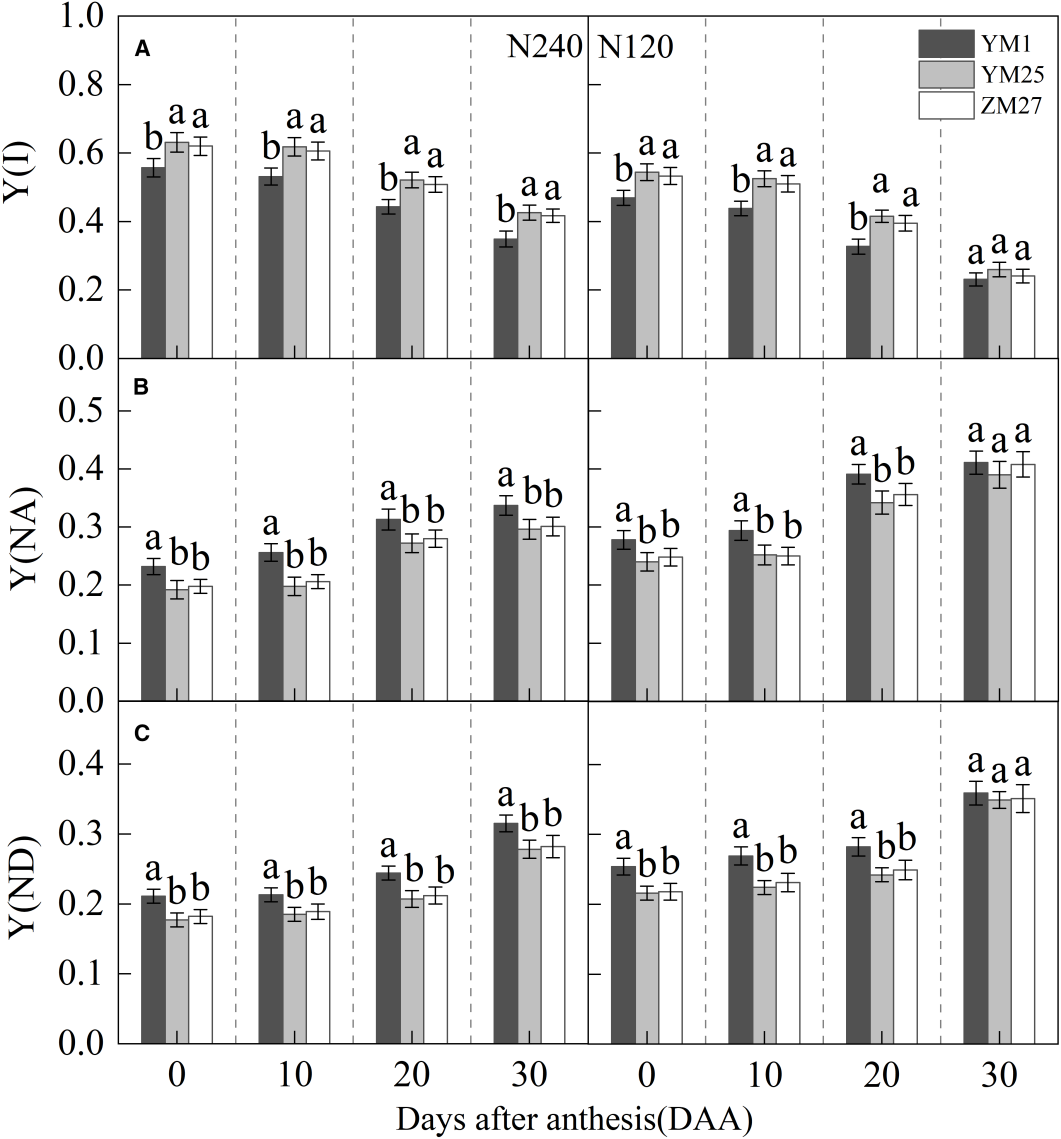
Differences of quantum yield of PSI effective quantum yield (Y(I)) **(A)**, non-photochemical quenching due to acceptor-side limitation (Y(NA)) **(B)**, non-photochemical quenching due to donor-side limitation (Y(ND)) **(C)** in flag leaves of wheat with different source-sink relationships. Notes: Each value represents the mean ± SD of three biological replicates. Different lowercase letters indicate statistically significant differences between treatments (*P < 0.05*) according to LSD test. Error bars indicate SD.

Y(I) gradually decreased with days after anthesis and decreased under lower N levels (**Figure 5A**). Under both N treatments, YM25 and ZM27 exhibited significantly higher Y(I) than YM1 during 0–20 DAA. Y(NA) and Y(ND) gradually increased with days after anthesis and increased with reduced N application (**Figures 5B, C**). Under both N treatments, Y(NA) and Y(ND) of YM25 and ZM27 were significantly lower than YM1 during 0–20 DAA. In contrast, YM1 showed a rapid increase in Y(NA) and Y(ND), reflecting limitations on both the acceptor and donor sides of PSI. This resulted in reduced light energy utilization efficiency at PSI in YM1, particularly under low N conditions. These findings suggest that YM25 and ZM27 can maintain more efficient PSI light energy utilization, with fewer limitations on electron transport, compared to YM1. Under low N conditions, Y(NA) and Y(ND) in all cultivars significantly increased, with a greater impact on Y(NA). This indicates that PSI receptor side blockage first occurs, causing a buildup of electrons on the receptor side due to PSI damage, leading to an increase in Y(NA). The downstream electron transfer of PSI is inhibited due to the lack of receptors, ultimately leading to a decrease in PSI light energy utilization efficiency.

### 3.5 Cyclic electron flow

**Figure 6:**
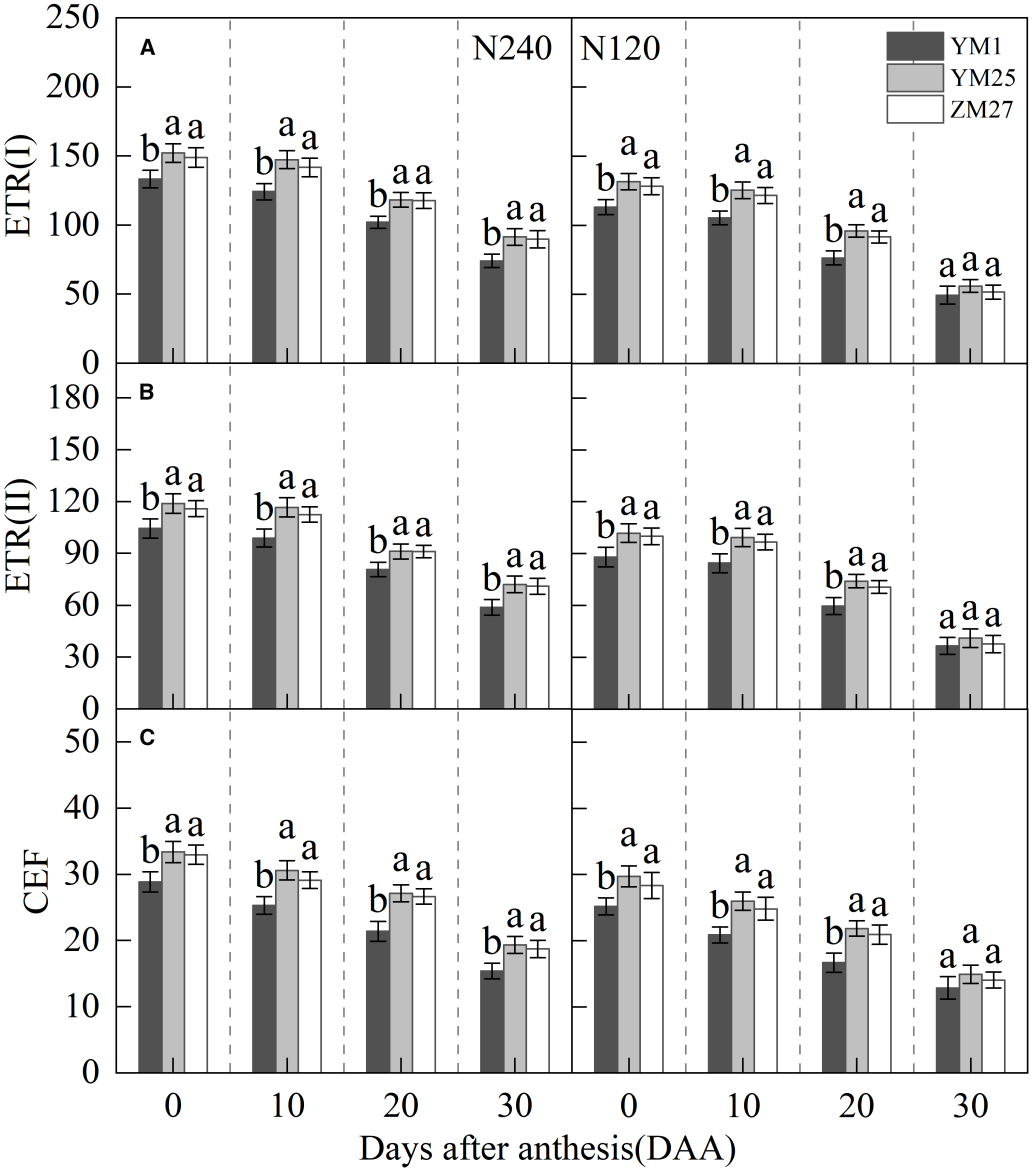
Differences of Electron transport rates ETR(I) **(A)**, ETR(II) **(B)** and cyclic electron flow **(CEF) (C)** in flag leaves of wheat with different source-sink relationships. Notes: Each value represents the mean ± SD of three biological replicates. Different lowercase letters indicate statistically significant differences between treatments (*P < 0.05*) according to LSD test. Error bars indicate SD.

ETR (I) and ETR (II) represent the electron transfer rates of PSI and PSII. CEF, a critical photoprotective mechanism that helps to alleviate photoinhibition. ETR(I), ETR(II), and CEF gradually decreased with days after anthesis and were suppressed under low N conditions (**Figures 6A–C**). YM25 and ZM27 exhibited significantly higher ETR(I), ETR(II), and CEF than YM1 during 0–20 DAA under both N treatments. No significant differences were found between YM25 and ZM27. Under low N conditions, ETR(I), ETR(II), and CEF in all cultivars significantly decreased. These results indicate that source-limited wheat can maintain higher electron transfer rates of PSI and PSII, effectively utilizing CEF to protect the photosynthetic system from light-induced damage. However, under low N conditions, the damage to the photosynthetic system was intensified, with reduced CEF, making photoinhibition more likely.

### 3.6 Soluble protein content

**Figure 7:**
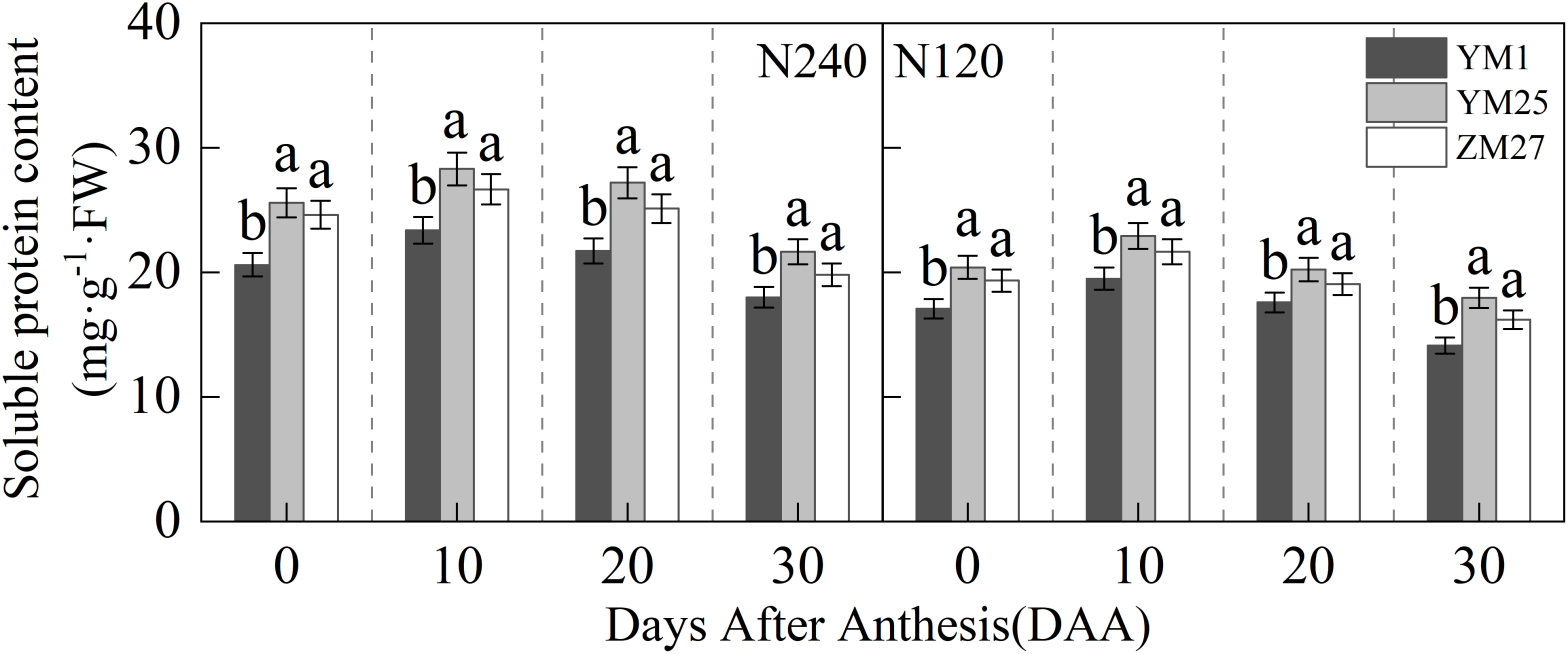
Differences of soluble protein content in flag leaves of wheat with different source-sink. Notes: Each value represents the mean ± SD of three biological replicates. Different lowercase letters indicate statistically significant differences between treatments (*P < 0.05*) according to LSD test. Error bars indicate SD.

The soluble protein content in the flag leaf first increased and then decreased with days after anthesis, and decreased under lower N levels (**Figure 7**). YM25 and ZM27 had significantly higher soluble protein content than YM1 during 0–30 DAA under both N treatments. No significant differences were observed between YM25 and ZM27. Under low N conditions, the soluble protein content in the flag leaves of all cultivars significantly decreased. These results indicate that source-limited wheat has higher soluble protein content, which can extend the photosynthetic functional period and delay leaf senescence.

### 3.7 Malondialdehyde content and O_2_^-^ production rate

**Figure 8:**
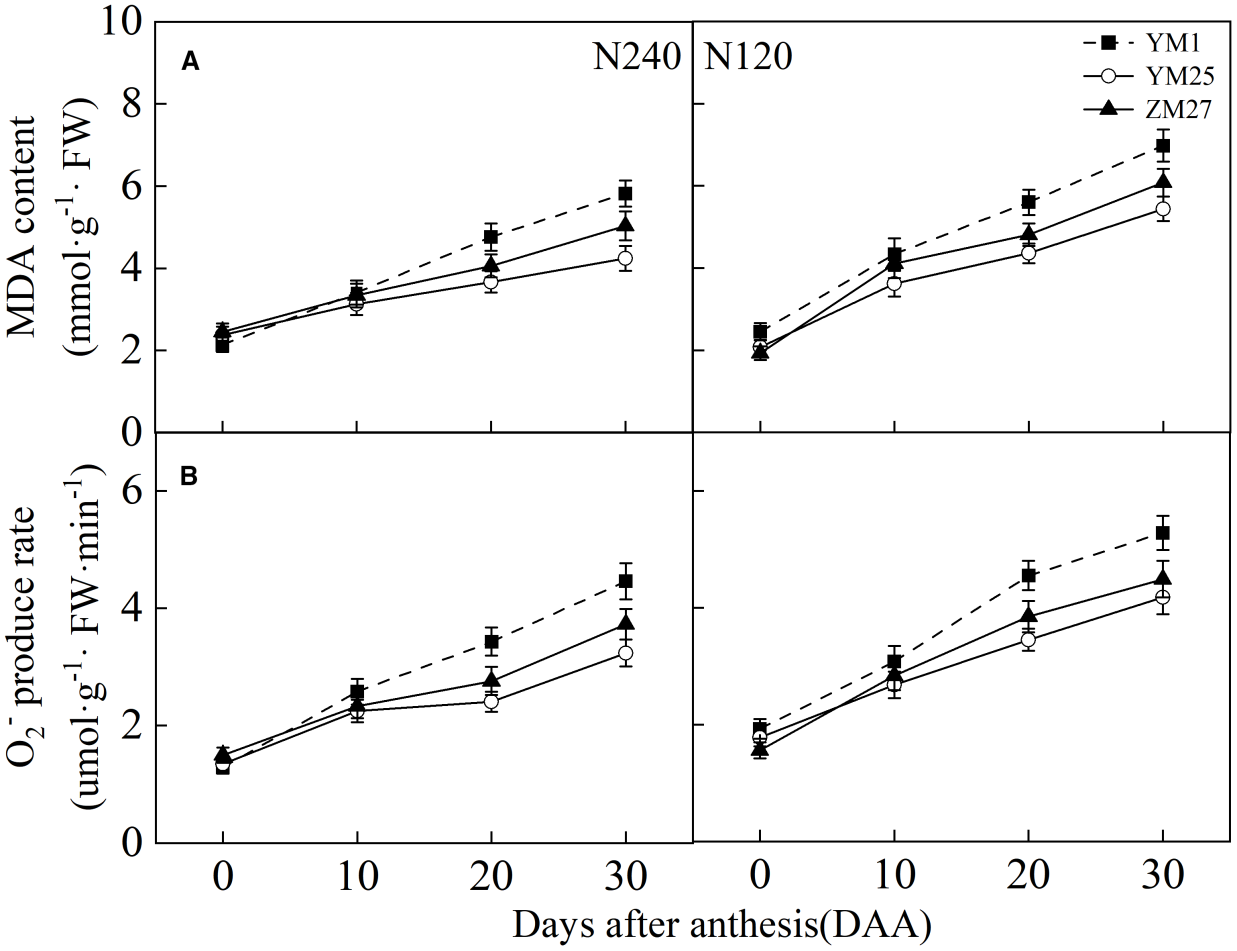
Differences of malondialdehyde (MDA) content **(A)** and O_2_^-^ production rate **(B)** in flag leaves of wheat with different source-sink relationships. Notes: Each value represents the mean ± SD of three biological replicates. Different lowercase letters indicate statistically significant differences between treatments (*P < 0.05*) according to LSD test. Error bars indicate SD.

MDA content and the O_2_^-^ production rate in the flag leaves increased with days after anthesis and were higher under low N conditions (**Figures 8A, B**). YM1 exhibited a faster increase in MDA content and O_2_^-^ production rate compared to YM25 and ZM27. During 20–30 DAA, YM25 and ZM27 had significantly lower MDA and O_2_^-^ levels than YM1, indicating less lipid peroxidation and oxidative stress. Under low N conditions, all cultivars exhibited a significant increase in ROS production, which led to greater lipid peroxidation. These findings highlight the superior oxidative stress tolerance of source-limited wheat, which helps to maintain leaf membrane stability and protect against photoinhibition.

### 3.8 Antioxidant enzyme activity

**Figure 9:**
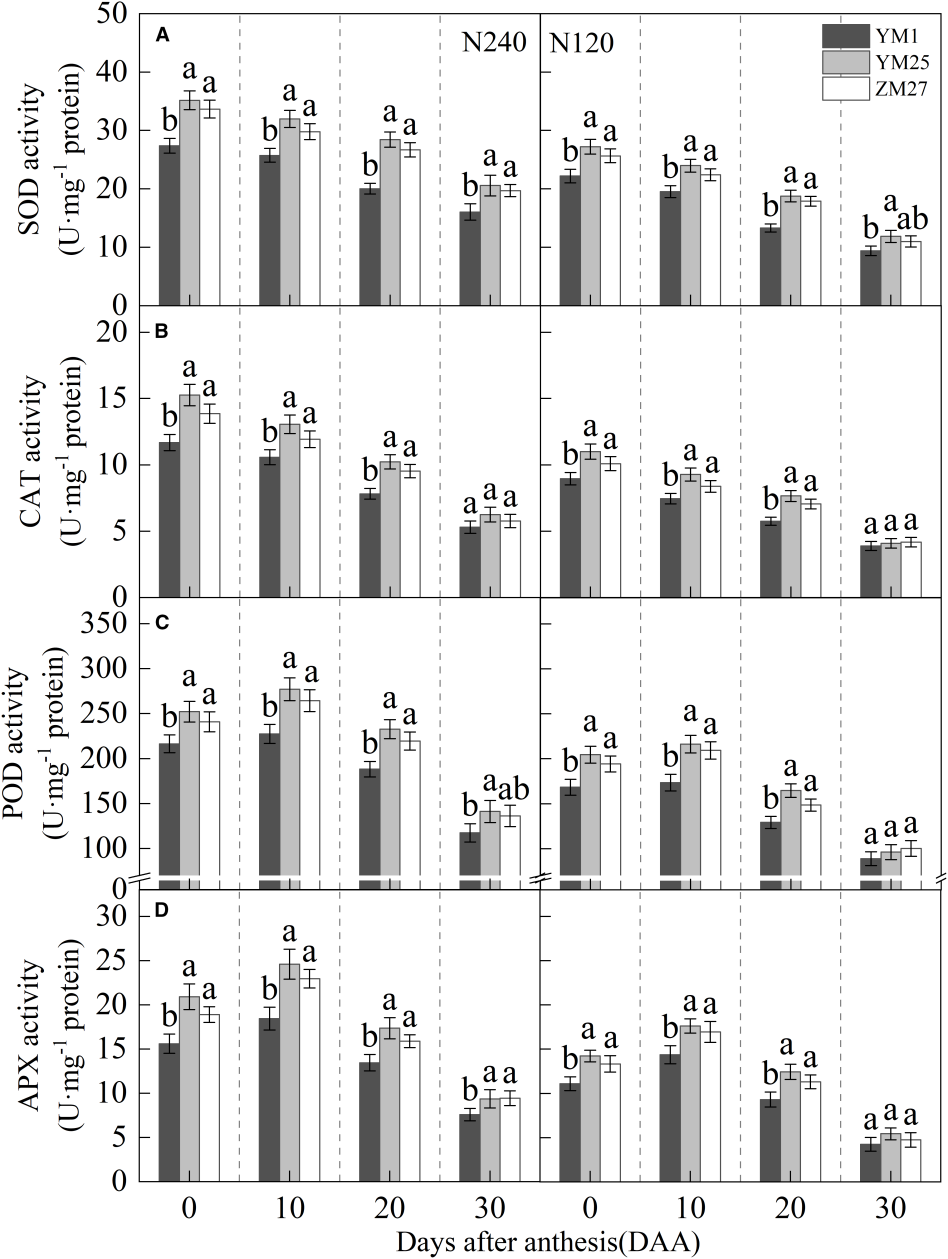
Differences of superoxide dismutase (SOD) activity **(A)**, catalase (CAT) activity **(B)**, peroxidase (POD) activity **(C)**, and ascorbate peroxidase (APX) activity **(D)** of wheat with different source-sink relationships Notes: Each value represents the mean ± SD of three biological replicates. Different lowercase letters indicate statistically significant differences between treatments (*P < 0.05*) according to LSD test. Error bars indicate SD.

The activity of SOD and CAT in the flag leaf gradually decreased with days after anthesis and decreased further with reduced N application (**Figures 9A, B**). The activities of POD and APX in the flag leaf first increased and then decreased with days after anthesis, reaching their maximum value at 10 DAA, and decreased under lower N levels (**Figures 9C, D**). Under N240 and N120 conditions, the activities of SOD, CAT, and APX in YM25 and ZM27 flag leaves were significantly higher than in YM1 during 0–20 DAA. The POD activity in YM25 and ZM27 flag leaves was significantly higher than in YM1 during 0–30 DAA. No significant differences were observed between YM25 and ZM27. These results suggest that source-limited wheat possesses stronger antioxidant defense systems, enabling it to scavenge reactive oxygen species more effectively and mitigate oxidative damage. Under low N conditions, antioxidant enzyme activities decreased across all cultivars, leading to increased oxidative stress and membrane damage.

## 4 Discussion

### 4.1 Photosynthetic capacity and source-sink relationships

Our previous study (Zhang et al., 2024a, 2025) explored source-sink relationships, yield, and photosynthetic differences among wheat cultivars, suggesting that the yield, kernels per spike, and thousand-kernel weight of source-limited wheat were significantly higher than sink-limited wheat. Previous studies also suggested that Rubisco activity, as well as sucrose synthesis and transport, regulate photosynthesis in wheat cultivars with different source-sink relationships. In our study, source-limited wheat (YM25 and ZM27) demonstrated higher photosynthetic capacity than sink-limited wheat (YM1), particularly in terms of chlorophyll content, *Pn_max_*, and light energy utilization efficiency (**Figures 2A**, **Figures 3A, B**). This finding aligns with previous research, which suggested that source-limited cultivars improved photosynthetic efficiency by enhancing light absorption and utilization (Zhang et al., 2024a, 2016). The higher chlorophyll content and *Pn_max_* in source-limited wheat suggest that these wheat types are more efficient at capturing solar energy and converting it into chemical energy, which is vital for sustaining high yield potential.

The greater photosynthetic capacity in YM25 and ZM27, especially under low N conditions, supports the hypothesis that optimizing the source-sink balance can alleviate the negative impacts of N limitation on photosynthesis. The efficient use of light energy and the enhanced ability of these cultivars to utilize solar radiation effectively are essential for maximizing photosynthetic efficiency and overall yield potential (Foyer and Noctor, 2005). These findings emphasize the importance of considering source-sink interactions in breeding strategies that are aimed at improving wheat productivity under varying environmental conditions.

### 4.2 Photoprotective mechanisms and light energy dissipation

Research has shown that photoinhibition is a common phenomenon in the late stage of wheat growth (Chai et al., 2022). NPQ and CEF play crucial roles in photoprotection by dissipating excess light energy and preventing photoinhibition (Derks et al., 2015; Yamamoto and Shikanai, 2019). Although NPQ exists in plants, it is one of the reasons for the potential decrease in crop yield due to its ability to reduce the quantum efficiency of photosynthesis (Christa et al., 2018). In this study, YM25 and ZM27 exhibited significantly lower NPQ and *Φ_NPQ_*, while *Φ_NQ_* was higher than YM1, indicating that these cultivars were more efficient at dissipating excess light energy without compromising photosynthetic efficiency (**Figures 4A–C**). The lower NPQ in source-limited wheat suggested that these cultivars maintained a more efficient light-energy dissipation system, allowing them to optimize photosynthetic performance and minimize light-induced damage (Murchie and Ruban, 2020; Muller et al., 2014). In contrast, YM1, with its sink-limited characteristics, exhibited higher NPQ and greater heat dissipation, which may reduce photosynthetic quantum efficiency and contribute to photoinhibition under high light conditions.

Analyzing the PSI photosynthetic quantum yield, Y(ND) represents the quantum yield of non-photochemical quenching in PSI caused by donor side limitation, while Y(NA) represents the quantum yield of non-regulated energy dissipation in PSI caused by acceptor side limitation. This study suggested that, compared with the sink-limited wheat YM1, the flag leaves of source-limited wheat YM25 and ZM27 had less limitation in the Y(NA) and Y(ND) of the PSI receptor and donor sides, maintaining stable downstream electron transport of PSI, resulting in higher PSI light energy utilization efficiency and electron transport capacity (**Figures 5B, C**). In contrast, the Y(NA) and Y(ND) of the flag leaves of sink-limited wheat YM1 increased significantly, blocking downstream electron transport in PSI, resulting in lower PSI light energy utilization efficiency.

The higher CEF in YM25 and ZM27 further supported the notion that source-limited wheat can better protect its photosystems from light-induced stress (**Figure 6C**). CEF is a crucial process for maintaining PSI light energy utilization efficiency and reducing oxidative damage to the photosynthetic apparatus (Yamamoto et al., 2021; Storti et al., 2019). CEF can alleviate excessive reduction and superoxide anion generation on the PSI receptor side, as well as protect PSI from photoinhibition (Cun et al., 2022). The ability to enhance CEF in response to light stress, particularly under low N conditions, highlights the photoprotective capacity of source-limited wheat and its ability to maintain stable photosynthetic performance despite environmental challenges.

The source-limited wheat YM25 and ZM27, characterized by a stronger sink demand relative to source capacity, help maintain a higher metabolic flux and delay the onset of senescence signals in the flag leaf. This sustained metabolic activity likely preserves the integrity and functionality of the photosynthetic apparatus for longer, enabling more efficient regulation of energy dissipation (lower wasteful NPQ, and *Φ_NPQ_*, higher regulated *Φ_NQ_*), facilitates greater CEF activity for balancing ATP/NADPH ratios and protecting PSI, and supports the sustained synthesis and activity of antioxidant enzymes and compounds, collectively leading to superior photoprotection. In contrast, the sink-limited wheat YM1 may lead to earlier feedback inhibition of photosynthesis and accelerated senescence, compromising the coordination of these photoprotective pathways and resulting in higher NPQ, lower CEF, and reduced antioxidant capacity, making it more susceptible to photoinhibition and oxidative damage.

### 4.3 Antioxidant defense and leaf senescence

Excess light energy in photosynthesis triggers reactive oxygen species (ROS) production via oxygen-dependent electron transfer (Szarka et al., 2012). This electron overload induces oxidative damage through lipid peroxidation and membrane disruption (Zaffagnini et al., 2012). Plants counteract this with dual antioxidant systems: low-molecular-weight compounds (e.g., glutathione) and enzymatic regulators (e.g., superoxide dismutase) (Zhu et al., 2023). Our study showed that source-limited wheat (YM25 and ZM27) exhibited higher levels of carotenoids, soluble proteins, and antioxidant enzyme activities (SOD, CAT, POD, and APX) compared to sink-limited YM1 (**Figure 2B**, **Figure 7**, and **Figures 9A–D**). The enhanced antioxidant capacity in these cultivars likely contributes to their superior ability to scavenge ROS and mitigate oxidative damage (**Figures 8A, B**), which is crucial for protecting the photosynthetic apparatus from photoinhibition and delaying leaf senescence (Ewald, 2018). The higher soluble protein content in YM25 and ZM27 suggests that these cultivars can sustain prolonged photosynthetic activity, which may delay leaf senescence and improve overall plant productivity (Zivcak et al., 2013).

In contrast, YM1, with its weaker source strength, exhibited a more rapid accumulation of ROS and MDA, indicating greater lipid peroxidation and membrane damage (**Figures 8A, B**). This accelerated oxidative stress and leaf senescence in YM1 may explain its lower photosynthetic capacity and yield potential compared to source-limited wheat (YM25 and ZM27). The ability of source-limited wheat to maintain higher antioxidant enzyme activity and reduce ROS production may be key to its superior environmental adaptability, allowing it to better cope with both high light and N-deficient conditions.

### 4.4 N fertilization and photosynthetic efficiency

N fertilization significantly improved the photosynthetic capacity and light energy utilization efficiency of all wheat cultivars, as reflected in higher *Pn_max_*, *Φ_CO2_*, and reduced photoinhibition under high N conditions (**Figures 3**-**5**). N is a critical nutrient for chlorophyll synthesis and enzyme function, and its availability directly influences photosynthetic efficiency (Mu and Chen, 2021). The results from this study confirmed that N fertilization alleviates the limitations imposed by low N levels, enabling plants to sustain higher *Pn* and better light energy utilization.

However, under low N stress, the leaves are more susceptible to light damage, and plants can alleviate photoinhibition by increasing heat dissipation (Pinnola and Bassi, 2018; Takahashi and Badger, 2011). Our study showed that under low N conditions, all cultivars exhibited decreased photosynthetic efficiency, increased photoinhibition, and greater oxidative stress (**Figures 3**-**5**, **Figure 8**). This highlights the importance of balanced N management to optimize photosynthesis and minimize yield losses due to nutrient deficiencies. While source-limited wheat (YM25 and ZM27) showed superior ability to mitigate the effects of low N through enhanced photoprotective mechanisms, N fertilization still played a crucial role in improving overall photosynthetic capacity and yield potential. Therefore, a combination of optimized N fertilization and source-sink balance management can significantly enhance wheat productivity.

### 4.5 Implications for wheat breeding and future research

This study provided valuable insights into the role of source-sink relationships and photoprotective mechanisms in improving wheat photosynthesis and yield potential. Source-limited wheat (YM25 and ZM27) demonstrated higher photosynthetic efficiency, greater resistance to photoinhibition, and superior antioxidant capacity compared to sink-limited wheat. These traits contribute to their better adaptability to environmental stresses, such as N limitation. The findings suggested that improving the source-sink relationship, along with optimizing N management, could significantly enhance wheat productivity, particularly in areas where N availability is limited.

Future research should focus on identifying the genetic pathways that regulate source-sink dynamics and photoprotective mechanisms in wheat. Understanding the molecular basis of these processes could provide new targets for breeding programs aimed at developing high-yielding wheat cultivars with improved photosynthetic efficiency and environmental adaptability. Additionally, investigating the interaction between source-sink relationships and other abiotic stresses, such as drought and heat, will be essential for developing wheat cultivars capable of withstanding the challenges posed by climate change.

## 5 Conclusion

Source-limited wheat, by enhancing the photosynthetic capacity of flag leaves, exhibited stronger light energy utilization efficiency and alleviated photoinhibition while reducing light-induced damage through decreased NPQ and increased CEF proportion. Additionally, source-limited wheat had stronger antioxidant capacity, which enabled it to scavenge reactive oxygen species, reduce membrane lipid peroxidation, and delay leaf senescence. Increased N fertilization significantly improved wheat’s photosynthetic capacity and light energy utilization efficiency, alleviating photoinhibition. Source-limited wheat maintained the integrity of the photosynthetic apparatus under low N conditions by enhancing photoprotective mechanisms, demonstrating stronger environmental adaptability. Therefore, proper N fertilization and optimization of the source-sink relationship can help improve wheat’s photosynthetic capacity and yield potential.

## Data Availability

The original contributions presented in the study are included in the article/supplementary material. Further inquiries can be directed to the corresponding author.
